# Recognition Memory is Improved by a Structured Temporal Framework During Encoding

**DOI:** 10.3389/fpsyg.2015.02062

**Published:** 2016-01-20

**Authors:** Sathesan Thavabalasingam, Edward B. O’Neil, Zheng Zeng, Andy C. H. Lee

**Affiliations:** ^1^Department of Psychology (Scarborough), University of Toronto, TorontoON, Canada; ^2^Rotman Research Institute, Baycrest Centre for Geriatric Care, TorontoON, Canada

**Keywords:** recognition memory, episodic memory, timing, temporal expectation, temporal structure, interference

## Abstract

In order to function optimally within our environment, we continuously extract temporal patterns from our experiences and formulate expectations that facilitate adaptive behavior. Given that our memories are embedded within spatiotemporal contexts, an intriguing possibility is that mnemonic processes are sensitive to the temporal structure of events. To test this hypothesis, in a series of behavioral experiments we manipulated the regularity of interval durations at encoding to create temporally structured and unstructured frameworks. Our findings revealed enhanced recognition memory (*d′*) for stimuli that were explicitly encoded within a temporally structured vs. unstructured framework. Encoding information within a temporally structured framework was also associated with a reduction in the negative effects of proactive interference and was linked to greater recollective recognition memory. Furthermore, rhythmic temporal structure was found to enhance recognition memory for incidentally encoded information. Collectively, these results support the possibility that we possess a greater capacity to learn and subsequently remember temporally structured information.

## Introduction

The human brain is adept at extracting regularities from the temporal dimension of experience. Given the ubiquity of repetitive phenomena and temporal patterns, the capacity to harness these temporal regularities allows for future outcomes to be anticipated, affording benefits to behavior and cognition. Indeed, it has been demonstrated that temporal regularities in stimulus presentation entrain attentional focus to their temporal structure, referred to as temporal expectation, which can confer perceptual and behavioral advantages (Jones and Boltz, 1989; Barnes and Jones, 2000; Jones et al., 2002, 2006; Nobre and Rohenkohl, 2014). Participants exhibit enhanced sensory processing and behavioral facilitation when stimuli are experienced at regular fixed interval durations compared to irregular temporal rhythms comprised of random or jittered interval durations (e.g., Olson and Chun, 2001; Jones et al., 2002, 2006; Lange, 2010; Mathewson et al., 2010; Rohenkohl and Nobre, 2011; Rohenkohl et al., 2012). These findings are supported by electrophysiological evidence suggesting that neural firing may vary dynamically as a function of the conditional probability that an event will occur at a particular time (Praamstra et al., 2006; Rohenkohl and Nobre, 2011). For instance, temporal expectation has been shown to modulate perceptual processing of visual stimuli through the entrainment of oscillatory activity in visual cortex (Cravo et al., 2013). Furthermore, temporal expectation has been suggested to combine with predictive information about other stimulus attributes such as location, leading to a top–down enhancement of perceptual processing in early visual areas (Doherty et al., 2005).

Although expectancies acquired through temporally structured events can modulate attentional, motor, and perceptual processes (Coull and Nobre, 2008; Nobre and Rohenkohl, 2014), it is unknown if similar benefits can be observed in other higher cognitive processes, in particular, episodic memory. There have, to our knowledge, been no systematic investigations of the relationship between temporal expectation and long-term memory processing. Although, previous work has explored the effects of inter-stimulus intervals (ISIs) on subsequent memory (e.g., Weaver, 1974; Lichtenstein and Keren, 1979; Proctor, 1983; Watkins, 1985), much of this research has focused on how the nature of ISIs can influence stimulus processing during learning such as rehearsal. Importantly, there are reasons to suggest that temporal expectation may impact episodic memory. Time is a fundamental component of episodic memory (Tulving, 1972) and the hippocampus, a key structure in episodic memory processing (Eichenbaum, 1999), has been implicated in the processing of temporal information. Human functional magnetic resonance imaging (fMRI) studies have provided evidence that the hippocampus is sensitive to contextual changes and the manipulation of ordinal item relationships (Ezzyat and Davachi, 2014; Hsieh et al., 2014), consistent with the view that item-driven changes in context may be important for linking events across time (Howard and Kahana, 2002). Beyond ordinal relationship manipulations, recent findings suggest that the hippocampus may be sensitive to temporal duration information. Rodent hippocampal CA1 cells have been observed to fire during the delay between two discontiguous events, potentially signaling the passage of time (Pastalkova et al., 2008; MacDonald et al., 2011), with comparable findings in monkeys (Naya and Suzuki, 2011). Moreover, human fMRI research has revealed that hippocampal activity is sensitive to temporal duration manipulations when ordinal item sequences are held constant, suggesting that events may be bound to a temporal framework (Barnett et al., 2014).

Here, we conducted a series of behavioral experiments to investigate the impact of expectations derived from temporally structured events on episodic memory. Since temporal expectation can enhance attentional and perceptual processes, we predicted that the encoding of stimuli within a temporally structured framework would result in enhanced memory performance. We tested this hypothesis in Experiment 1, where participants remembered scene images in the context of a yes/no recognition task. Participants completed two study-test blocks, one with a regular and one with an irregular temporal structure during study. Critically, this manipulation was unbeknownst to participants. Experiment 2 explored how this temporal structure might protect against proactive interference in a between-subject investigation of task order (temporally structured encoding followed by temporally unstructured encoding, and vice versa). Confidence judgments were also collected to investigate the impact of temporally structured encoding on recollective (i.e., remembering) and familiarity (i.e., feeling of knowing) memory. Finally, Experiment 3 examined whether an advantage for temporally structured stimulus presentation could be demonstrated with incidental, as opposed to intentional, stimulus encoding.

## Experiment 1

### Participants

Sixteen participants (mean age = 18.56 years; *SD* = 1.26; 12 female) took part in Experiment 1. Since there is, to our knowledge, no previous work examining the impact of temporal expectation on recognition memory, we had no prior knowledge of the magnitude and variability of expected effect sizes. As such, we made an *a priori* decision to have a participant group of 16 in order to have two participants for each of the eight counterbalanced versions that were created for the experimental tasks. Notably, our group size is consistent with previous studies examining the effects of temporal expectation on other cognitive domains (e.g., 13–16 subjects; Jones and Boltz, 1989; Correa and Nobre, 2008; Rohenkohl et al., 2012). All participants for this and subsequent experiments were undergraduate students recruited from the University of Toronto, Scarborough Campus, and were compensated for their time with course credit. Participants provided written informed consent prior to participation and all experiments were approved by the University of Toronto Research Ethics Board (ref. 26827).

### Procedure

All experimental tasks were completed on a desktop computer running E-Prime 2.0 software (Psychology Software Tools, Inc.), and displayed on a 19-inch monitor with a spatial resolution of 1024 × 768 pixels. Stimuli consisted of grayscale images (350 × 350 pixels) of a variety of real-world scenes (e.g., outdoor landscapes, indoor rooms, buildings, etc.) presented centrally on the screen with a black background.

**FIGURE 1:**
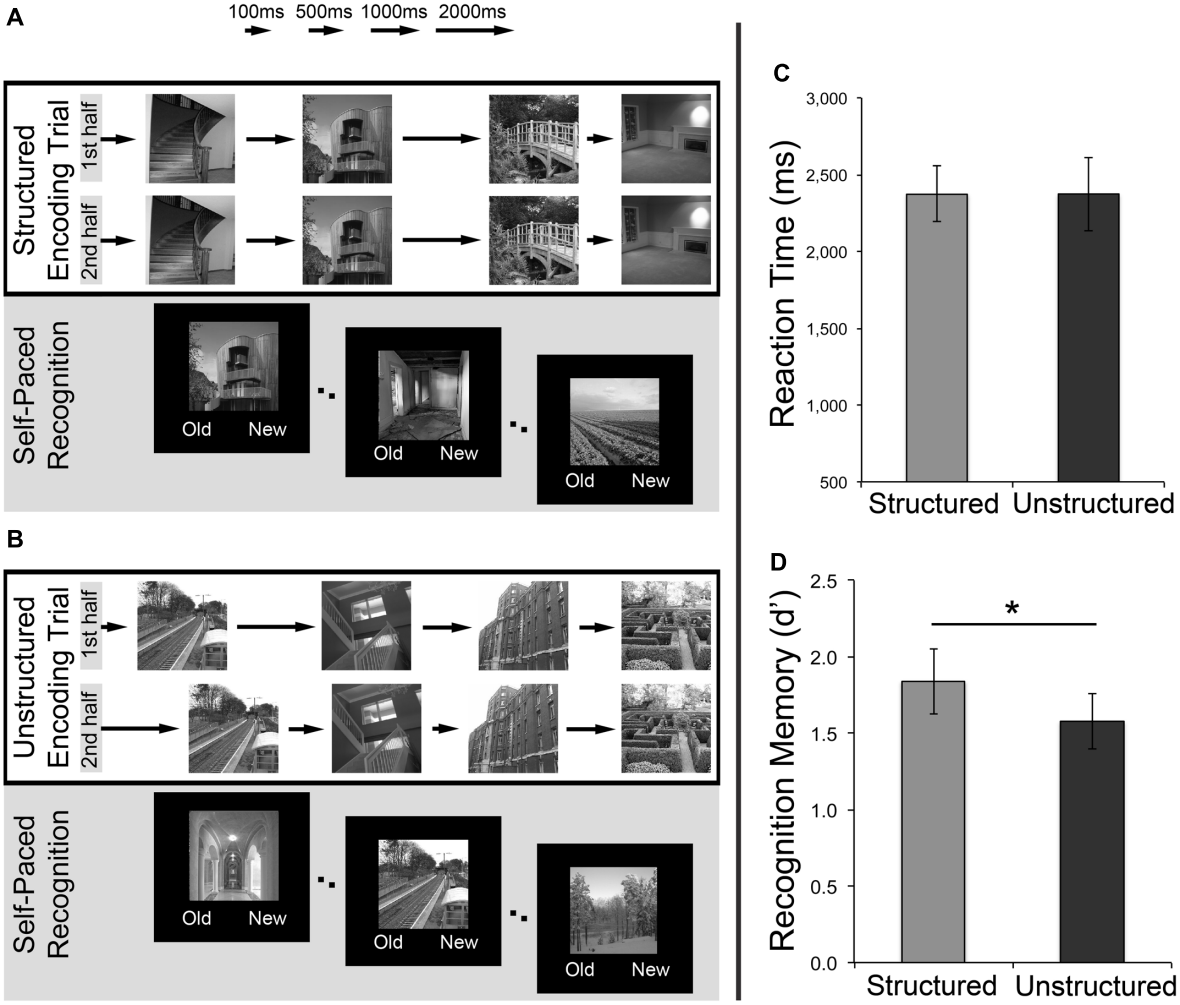
**Investigating the effects of temporally structured and unstructured learning on recognition memory. (A)** An example of a trial from the structured encoding task. A sequence of four images (each displayed for 700 ms) was shown twice in succession, separated by a sequence of intervals (500, 1000, 2000, 100 ms) that possessed an underlying temporal structure. **(B)** An example of a trial from the unstructured encoding task. A sequence of four images (each displayed for 700 ms) was shown twice in succession, with no regularity to the ordering of temporal intervals separating the image presentations. Following the study phase for both tasks, participants completed a self-paced recognition memory task for the studied images intermixed with a set of novel stimuli. **(C)** Mean reaction times during recognition test for temporally structured and unstructured tasks. **(D)** Subsequent recognition memory performance (measured using *d′*) for scenes presented in temporally structured and unstructured tasks. Error bars represent standard error of the mean. ^∗^*p* < 0.05.

In Experiment 1, participants completed two recognition memory tasks in a repeated-measures design, one temporally structured, and another temporally unstructured. Each recognition memory task consisted of an initial study phase followed immediately by a test phase (**Figures 1A,B**). In both tasks, participants were explicitly instructed to remember the images for a later recognition memory task. During the study phase, 48 unique grayscale scene images were presented individually for 700 ms to the participant. Scene presentation was segmented into mini-sequences of four, with each mini-sequence being shown twice in succession (e.g., A-B-C-D – A-B-C-D – E-F-G-H – E-F-G-H – etc.) in order to create a sense of predictability and maximize expectations as a consequence of temporal duration structure. Specifically, in the ‘temporally structured’ task (**Figure 1A**), the ISIs that followed the four images in each mini-sequence during study were presented in a repeating pattern across trials (i.e., 500, 1000, 2000, and 100 ms), giving rise to a regular temporal structure (e.g., A1-B2-C3-D4 – A1-B2-C3-D4, with letters referring to images, and numbers referring to interval durations). Conversely, in the ‘temporally unstructured’ task (**Figure 1B**), the ISIs did not possess any underlying temporal structure. Rather, four pseudo-randomly ordered ISIs followed the four stimuli within each mini-sequence shown during study. In order to match average ISI length to the temporally structured condition, these four ISIs (which were trial-unique) were derived from jittering around mean durations of 100 ms (*SD:* 40 ms), 500, 1000, and 2000 ms (all *SD*: 80 ms). The order of these ISIs for each mini-sequence was also randomized such that the ordinal position of each ISI differed during the initial and subsequent presentation (e.g., A1-B3-C4-D2 – A2-B4-C2-D1). Importantly, while participants were instructed to explicitly encode the stimuli, they were not made aware of the difference in ISI structure between the two recognition memory tasks. This incidental acquisition of temporal regularity was facilitated by using a sequence of ISIs, as opposed to a single, fixed ISI in the temporally structured task, thereby minimizing the likelihood that individuals would recognize the interval duration structure and focus attention on time.

During each test phase, participants completed a self-paced yes/no recognition task. Participants were presented with 48 images from the respective study phase, intermixed with 48 novel scene images and were required to indicate whether each scene was ‘old’ or ‘new’ using pre-specified keyboard presses. Target presentation was pseudo-randomized to minimize recency effects, with test stimuli being presented in the same quartile of trials as presented at study. The order of the recognition memory tasks was fully counterbalanced across participants (i.e., eight subjects received the temporally structured recognition memory task followed by the temporally unstructured recognition memory task, and eight subjects received the reverse order) and images were never repeated across the two tasks. In addition, targets and foils, as well as the assignment of each image to the structured or unstructured tasks, were counterbalanced across subjects.

### Results

Mean reaction times (RTs) for recognition memory decisions (**Figure 1C**) did not differ significantly between the temporally structured and unstructured tasks, as revealed by a paired-samples *t*-test [*t*(15) = 0.003, *p* > 0.250]. The proportion of targets correctly identified as “old” (i.e., Hits, H), and the proportion of foils incorrectly identified as “old” (i.e., false-alarms, FA), was calculated for each participant. In accordance with signal detection theory (Snodgrass and Corwin, 1988) these values were used to calculate a d-prime (*d′*) score (Z(P[H]) – Z(P[FA])), as a measure of sensitivity/recognition memory accuracy. Recognition performance (*d′*) for the studied images was above chance for items studied in both the structured [*t*(15) = 8.56, *p* < 0.001 two-tailed] and the unstructured [*t*(15) = 8.75, *p* < 0.001 two-tailed] conditions. Critically, assessment of the effect of temporal structure on recognition revealed significantly higher *d′* estimates for temporally structured recognition as compared to temporally unstructured recognition [*t*(15) = 2.21, *p* = 0.043 two-tailed, Cohen’s *d* = 0.55]. Thus, consistent with the prediction that expectations derived from temporal regularity in encoding can impact mnemonic processing, scenes studied within a temporally structured framework (comprising a repeating sequence of ISI durations), resulted in superior recognition memory (**Figure 1D**).

As stimuli studied during a structured encoding phase were embedded within a repeating sequence of ISIs, a proportion of stimuli were consistently followed or preceded by shorter ISIs (e.g., 100 ms), whereas others were followed or preceded by longer temporal intervals (e.g., 2000 ms). The observed subsequent memory advantage for the temporally structured task may, therefore, have arisen due to the consistently longer post-presentation processing time for a number of stimuli (thus allowing more time for consolidation into memory) or more preparation time, which was not available in the temporally unstructured encoding phase when the ordinal position of each ISI differed between initial and subsequent mini-sequence presentations. To assess this possibility, the average proportion of recognition hits for the temporally structured task was computed separately for the 1st, 2nd, 3rd, and 4th stimulus within each mini-sequence. This allowed us to assess any potential differences in recognition hits for stimuli that were followed or preceded by each of the four ISIs (500, 1000, 2000, 100 ms) during the study phase. A one-way repeated-measures ANOVA revealed no significant differences in recognition hits between the four stimuli within each mini-sequence [*F*(3,45) = 0.314, *p* = 0.815]. This finding demonstrates that memory performance was not mediated by differences in the length of the ISIs that followed/preceded each image, and precludes the possibility that the mnemonic advantage demonstrated in the temporally structured recognition task was driven by differences in consolidation or preparation time. Instead, we interpret this superior recognition accuracy as a product of the overall rhythmic temporal pattern that the scene images were presented in at encoding.

## Experiment 2

In Experiment 2, we aimed to confirm the findings in Experiment 1 through replication. Additionally, we wanted to examine if there were between-subjects differences in memory as a function of task order and if there were qualitative differences reflected in the confidence of participants’ recognition judgments (which were not collected in Experiment 1). It is conceivable that the order in which participants completed the two memory tasks in Experiment 1 (temporally structured followed by unstructured, or vice versa) impacted performance. Exposure to and retrieving an increasing number of items can result in proactive interference, in which memory for new items is negatively affected by previously encoded information (Criss and Shiffrin, 2004; Criss et al., 2011). In the present experimental design, therefore, proactive interference may reduce memory accuracy at the second recognition test phase, in particular during the latter trials. One question is whether temporally structured encoding mitigates this effect, resulting in a mnemonic advantage at the second test phase. A related second question is how temporally structured encoding qualitatively effects recognition memory. Recognition memory is widely believed to consist of recollection and familiarity (Mandler, 1980) and diminished effects of proactive interference have been suggested to be the result of a qualitative shift toward a greater reliance on recollection (Jacoby et al., 2010, 2013). We were interested, therefore, in whether a mnemonic advantage in the second test phase could be reflected in increased recollection.

### Participants

While we were interested in a within-subjects effect in Experiment 1 (temporally structured vs. unstructured encoding), we were focused on a between-subjects effect in Experiment 2, in particular an interaction between task order (order 1 = structured followed by unstructured vs. order 2 = unstructured followed by structured) and condition (structured vs. unstructured). To achieve satisfactory power for this interaction we made an *a priori* decision to increase our sample size from 16 in Experiment 1 to 44 in Experiment 2. To achieve this, 45 participants were recruited and took part in Experiment 2 (mean age = 19.64 years; *SD* = 2.76; 35 female). One participant was excluded for failing to comply with task instructions, leading to improper use of the confidence scale, resulting in our final sample of 44 (mean age = 19.66 years; *SD* = 2.79; 34 female). Notably, a *post hoc* power analysis using the effect size we observed for our interaction of interest (see Experiment 2 Results), reveals that our mixed effects ANOVA was adequately powered with our chosen sample size (>90%).

### Procedure

The task in Experiment 2 was identical to Experiment 1 except that a two-step response procedure was used, allowing collection of confidence ratings during the recognition phase of the structured and unstructured tasks. For every image, participants were required to indicate using a keyboard press whether the scene was old or new. Next, they indicated the confidence level of their response using a 1 to 3 scale: 1 – “*Very sure*”, 2 – “*Somewhat sure*”, and 3 – “*Not sure*” (i.e., total of six possible responses). Participants were instructed to give “*Very sure*” responses if they remembered the item, and were consciously aware of specific details associated with the study episode. In contrast, if they remembered seeing the scene image but could not bring to mind specific details of the study episode, they were asked to give a “*somewhat sure*” response. Finally, “*Not sure*” responses were provided if participants were unsure that the target scene image was old/new. Participants were encouraged to make full use of the entire confidence scale.

### Results

**FIGURE 2:**
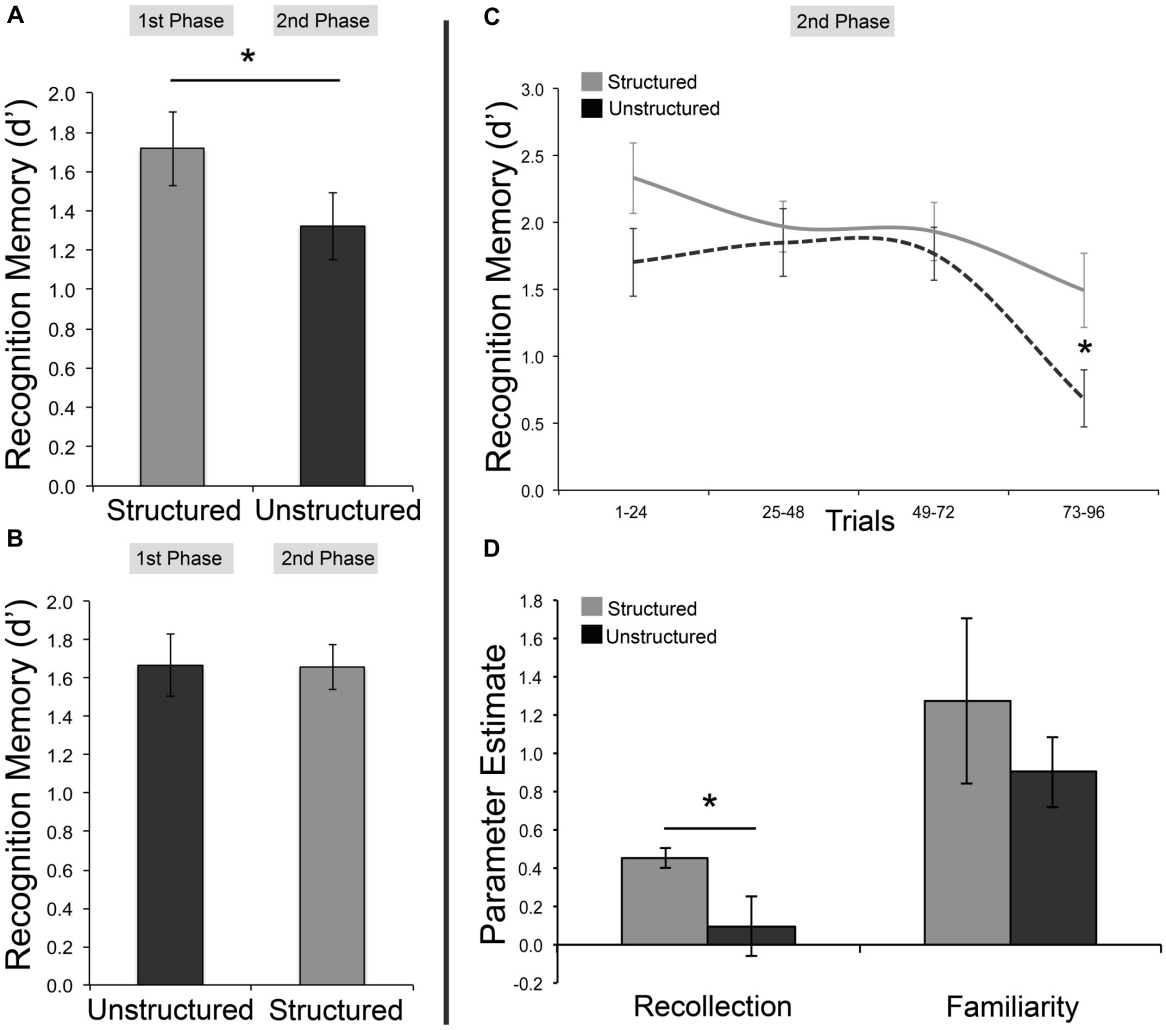
**Examining the effects of proactive interference on temporally structured and unstructured learning.** Results from both first and second test phase (left), and from second test phase only (right). **(A)** Recognition memory accuracy (as measured by *d′*) for individuals completing temporally structured encoding and test, followed by unstructured encoding and test. **(B)** Recognition memory accuracy for individuals completing temporally unstructured encoding and test, followed by structured encoding and test. **(C)** Recognition memory performance as a function of trial bin, during the second test phase for structured and unstructured tasks. **(D)** Parameter estimates of recollection and familiarity for temporally structured and unstructured tasks, during the second test phase. Error bars represent standard error of the mean. ^∗^*p <* 0.05.

First, we assessed whether the critical finding of Experiment 1, a mnemonic benefit for stimuli studied with a temporally structured presentation, could be replicated. A benefit for temporally structured encoding was indeed observed, with greater subsequent memory (*d′*) for scenes embedded within a temporally structured as opposed to temporally unstructured framework [*t*(43) = 2.21, *p* = 0.032 two-tailed, Cohen’s *d* = 0.33, structured *M* = 1.69, 95% *SEM =* 0.12, unstructured *M* = 1.49, *SEM* = 0.11]. Average RTs did not differ between temporally structured and unstructured recognition [*t*(43) = 0.102, *p* > 0.250 two-tailed]. To examine the effect of task order on memory performance, *d′* estimates were entered into a 2 × 2 mixed-effects ANOVA, with order (order 1 = structured followed by unstructured; order 2 = unstructured followed by structured) as a between-subjects factor, and condition (structured vs. unstructured encoding) as a within-subjects factor. A main effect of condition [*F*(1,42) = 5.68, *p* = 0.022, $\eta_{p}^{2}$ = 0.119] was found, as well as a significant condition by order interaction [*F*(1,42) = 6.03, *p* = 0.018, $\eta_{p}^{2}$ = 0.126]. In line with our expectation that proactive interference should result in a decline in performance at the second test phase, follow-up pairwise comparisons revealed a significant drop in accuracy between the first and second recognition test for order 1 [structured, followed by unstructured; *t*(22) = 3.17, *p* = 0.004 two-tailed, Cohen’s *d* = 0.66]. This decline, however, was not observed for order 2 [unstructured, followed by structured; *t*(20) = 0.056, *p* > 0.250 two-tailed; **Figures 2A,B**], and is consistent with the idea that encoding stimuli within a temporally structured framework can mitigate adverse effects on memory due to a build-up of proactive interference.

The negative effects of proactive interference were expected to be greatest at later trials of test. Hence, we hypothesized that the greatest differences in *d′* scores between the temporally structured and unstructured task should be observed at these later trials in the second test phase. To explore this, the 96 trials for the second test phase were divided equally into four 24-trial bins (bin 1 = trials 1–24; bin 2 = trials 25–48; bin 3 = trials 49–72; bin 4 = trials 73–96). Separate *d′* scores were computed for each bin, providing the opportunity to examine changes in memory performance as the test phase of the second recognition task progressed (**Figure 2C**). Average *d′* estimates for each trial bin were explored using a 2 × 4 mixed-effects ANOVA, with condition as a between-subjects factor, and trial bin as a within-subjects factor. A significant main effect of trial bin was found [*F*(3,126) = 11.88, *p* < 0.001, $\eta_{p}^{2}$ = 0.220]. Consistent with our prediction that memory performance would be negatively affected as testing progressed, a regression model fit to the data revealed a linear decline of mean *d′* scores (β = -0.283, *p* < 0.001] from trial bin 1 to 4 (bin 1: *M* = 2.02, *SEM =* 0.18; bin 2: *M* = 1.91, *SEM =* 0.16; bin 3: *M* = 1.85, *SEM =* 0.15; bin 4: *M* = 1.09, *SEM =* 0.17). Although the interaction between condition and trial bin was not significant [*F*(3,126) = 1.93, *p* = 0.128, $\eta_{p}^{2}$ = 0.044], planned one-tailed, independent-samples comparisons revealed a significant difference in accuracy between temporally structured and unstructured recognition in the fourth trial bin [*t*(42) = 2.34, *p* = 0.011, Cohen’s *d* = 0.70], but not the first, second, or third bins (all *p*’s ≥ 0.045; Bonferroni corrected critical *p* = 0.012).

Parameter estimates of recollection and familiarity were computed for the second test phase to evaluate if the mnemonic advantage for items encoded within a temporally structured framework emerging at the second test phase may also be reflected in distinct memory processes. These estimates were derived by fitting the dual process signal detection model (DPSD; Yonelinas, 1994) to receiver operating characteristic curves (ROCs) plotted using the confidence judgments for each subject for the temporally structured and unstructured tasks. In brief, the DPSD model is a hybrid threshold and equal variance signal detection model of recognition memory, and uses a sum-of-squares search algorithm to compute measures of recollection and familiarity (represented as a measure of discriminability, *d′*; http://yonelinas.faculty.ucdavis.edu/software/). An independent-samples *t*-test demonstrated that recollection memory was significantly lower for temporally unstructured, compared to structured recognition [*t*(42) = 2.108, *p* = 0.041 two-tailed, Cohen’s *d* = 0.65] in the second test phase (**Figure 2D**). No significant differences in familiarity were found between structured and unstructured recognition [*t*(42) = 0.814, *p* = 0.42 two-tailed], although notably the former was numerically greater than the latter.

Finally, debriefing data were collected for a majority of the participants (70%) to determine if they were aware of any differences in the temporal structure of the stimulus presentation at encoding. Of these, only one subject identified a difference in temporal structure between the encoding phases of the two tasks, suggesting that the observed benefit of temporally structured encoding to subsequent recognition memory is unlikely to be dependent on explicit awareness of the underlying rhythmic framework of stimulus presentation.

To summarize, it was found that the order in which individuals completed the first and second recognition task did indeed have an effect on memory performance. Specifically, performing the temporally unstructured, but not structured, task second was associated with a significant decrease in performance. The observed preservation of memory performance for temporally structured recognition in the second test phase suggests that items encoded within a temporally structured framework may be more resistant to proactive interference caused by the accumulation of prior learning. This may be reflected by a greater ability for individuals to leverage contextual information at retrieval, as indicated by the significant difference in recollection memory during the second test phase.

## Experiment 3

Participants intentionally learned stimuli in Experiments 1 and 2, with implicit processing of the underlying temporal structure in which items were presented. Interestingly, studies have demonstrated that implicit and explicit timing tasks potentially engage discrete neuroanatomical substrates (Coull and Nobre, 2008). Moreover, previous research has demonstrated that incidental learning of an ordinal sequence of finger movements can be facilitated by a regular temporal structure (O’Reilly et al., 2008). Experiment 3, therefore, examined whether the temporal structure present in an explicit timing task (in which participants were instructed to attend to durations) could also impact subsequent memory performance for items that were encoded incidentally.

### Participants

As in Experiment 1, we were investigating a within-subjects effect between structured and unstructured temporal frameworks in Experiment 3. Subsequently, to be consistent with our first experiment, we made an *a priori* decision to have 16 participants in Experiment 3. To achieve this, 19 undergraduate students were recruited and tested (mean age = 19.67 years; *SD* = 4.72; 15 female). One participant was excluded due to negative *d′* scores for both temporally structured and temporally unstructured subsequent recognition memory, and two additional participants misunderstood task instructions resulting in an inappropriate use of button responses, leaving a final sample of 16 (mean age = 19.88; *SD* = 4.36, 12 female).

### Procedure

**FIGURE 3:**
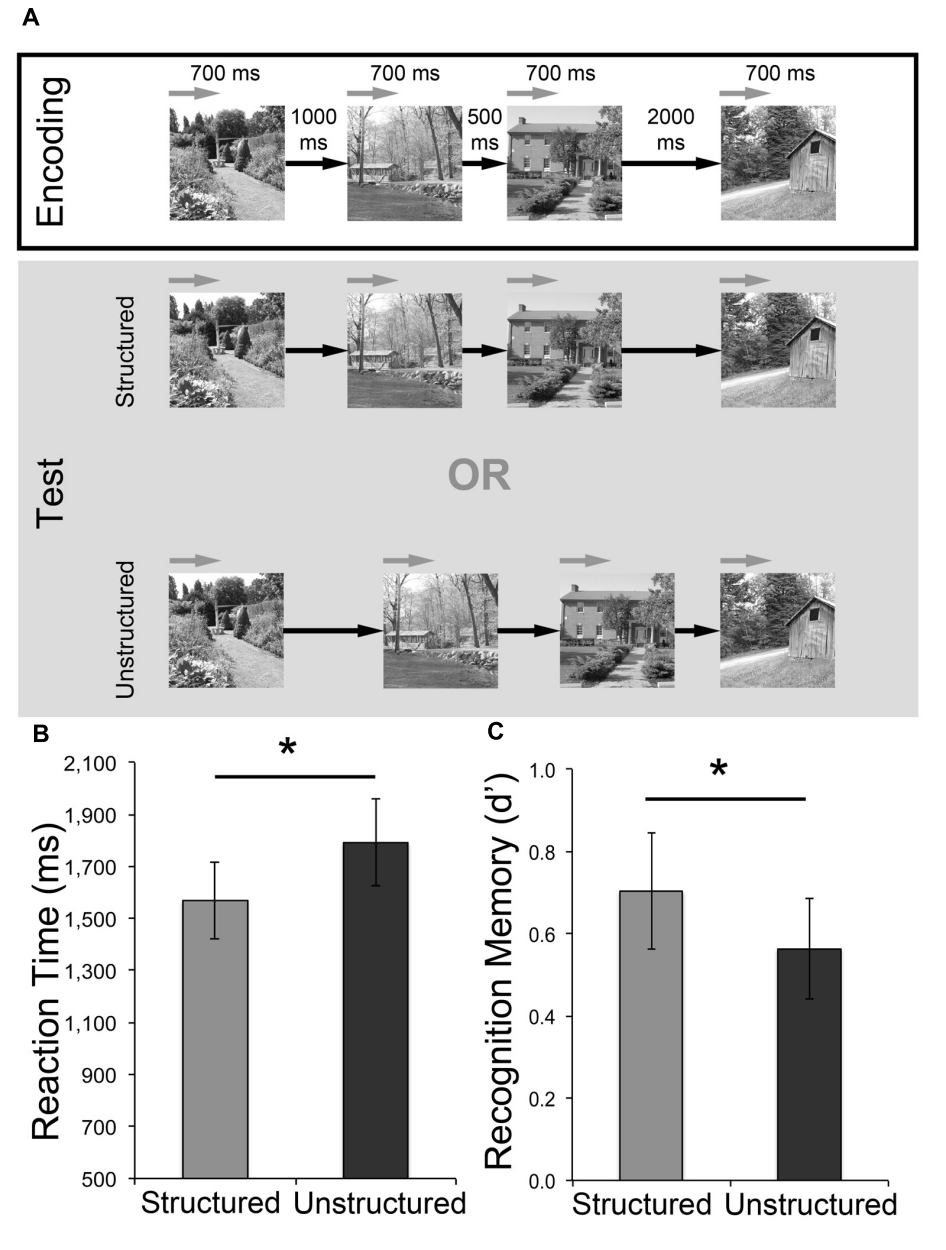
**Effect of temporally structured and unstructured learning for incidentally encoded stimuli. (A)** Participants were presented with trials in which they were instructed to monitor interval durations and make a match vs. mismatch decision. In the encoding phase of each trial participants saw four scenes separated by three intervals (mean: 500, 1000, 2000 ms). This was followed by a test phase, where the four scenes were repeated in the same order with their original interval durations (structured) or the rearranged interval durations (unstructured). The encoding and test phases were separated by a 3500 ms fixation cross, and participants were asked to indicate their response during a 2500 ms response screen showing the words “Change (1) or No Change (2)?” at the end of each test phase. **(B)** Mean reaction times for memory responses during the surprise recognition test. **(C)** Memory performance (as measured by *d′*) for the surprise recognition test. Error bars represent standard error of the mean. ^∗^*p* < 0.05.

Each trial consisted of a study, test, and response phase (**Figure 3A**). The study phase comprised of four scene images presented sequentially for 700 ms each. These scenes were separated by three blank ISIs, jittered around mean durations of 500, 1000, and 2000 ms (all *SD*: 80 ms), with the order of these ISIs pseudo-randomized across trials. This was followed by a 3500 ms fixation cross, and the re-presentation of the four scenes (in the same order) in a subsequent test phase. As with the previous experiments, the nature of the ISIs distinguished temporally structured and unstructured trials. For temporally structured trials, the ISI durations in repeated sequences were maintained, whereas in temporally unstructured trials, the ISIs were rearranged completely. Immediately after each test phase, participants would indicate if there was “No change” or “Time change” during a 2500 ms response screen. Participants completed six trials each of these structured and unstructured time judgments (48 total image presentations), and temporally structured and unstructured trials were pseudo-randomized. Following completion of these trials, a surprise self-paced recognition memory task was administered. The 48 studied images and 48 intermixed foil images were presented, one at a time. Participants indicated whether each image was old or new using a keyboard.

### Results

There was no significant difference in accuracy [*t*(15) = 0.298, *p* > 0.250] in the detection of structured (*M* = 0.81, *SEM* = 0.04) vs. unstructured (*M* = 0.79, *SEM* = 0.06) intervals in the incidental encoding task, although participants demonstrated significantly slower response times [*t*(15) = 2.34, *p* = 0.033 two-tailed; Cohen’s *d* = 0.59] for unstructured (*M* = 936 ms, *SEM* = 92.84) compared to structured (*M* = 775 ms, *SEM* = 74.79) temporal interval trials. For the surprise recognition task, average RTs were also significantly slower for scenes that were incidentally encoded in temporally unstructured, compared to structured, frameworks [*t*(15) = 2.169, *p* = 0.047 two-tailed, Cohen’s *d* = 0.54; **Figure 3B**]. Examination of *d′* scores revealed above chance recognition memory performance for both conditions [structured: *t*(15) = 5.00, *p* < 0.001 two-tailed; unstructured: *t*(15) = 4.59, *p* < 0.001 two-tailed]. Critically, as predicted in light of the *d′* findings in Experiments 1 and 2, recognition memory was significantly greater for structured than unstructured conditions [*t*(15) = 1.96, *p* < 0.034 one-tailed, Cohen’s *d* = 0.49; **Figure 3C**]. Average *d′* scores in Experiment 3 were noticeably lower than the previous two experiments. However, this finding is not surprising given that participants were not explicitly told to study and remember the scene stimuli. Overall, these results demonstrate that the rhythmic temporal structure of items perceived in trials with consistent interval durations between study and test repetitions can confer mnemonic advantages, even when subjects are attending to the intervals and not the items themselves (see **Table 1** for summary of key findings across all three experiments).

**Table 1:** Mean recognition memory accuracy (*d′*) and reaction times (RTs) for the temporally structured and unstructured tasks in all three experiments (*SEM* are reported in parentheses).

		Reaction time (ms)		Recognition memory (*d′*)	
Experiment	Sample	Structured	Unstructured	Structured	Unstructured
1	16 (12 Female)	2374 (180.24)	2374 (239.69)	1.84^∗^ (0.21)	1.58 (0.18)
2	44 (34 Female)	3846 (177.50)	3830 (182.57)	1.69^∗^ (0.12)	1.48 (0.11)
3	16 (12 Female)	1569^∗^ (149.19)	1792 (165.83)	0.70^∗^ (0.14)	0.56 (0.12)

*An asterisk indicates a significant difference between structured and unstructured tasks at *p* < 0.05.*

## Discussion

Across three behavioral experiments, we have demonstrated that learning within a structured temporal framework can enhance subsequent recognition memory. Our findings converge with work that has suggested that temporal regularities can facilitate perceptual processing and motor responding (Nobre and Rohenkohl, 2014). Importantly, however, we provide novel evidence that the cognitive advantages resulting from temporally structured stimulus presentation can be extended from perceptual or attentional enhancements occurring on a relatively short time scale (e.g., during exposure to a continuous stream of stimuli) to mnemonic benefits occurring over longer time scales (e.g., recognition memory for the individual items from a stream of stimuli). Experiment 1 demonstrated that intentionally encoding items within a structured temporal framework enhanced subsequent recognition memory (as measured by *d′*) compared to learning within an unstructured temporal framework. Given that participants completed both temporally structured and unstructured recognition tasks, Experiment 2 used a between-subjects design to investigate the potential impact of task order. We found a particular mnemonic advantage for encoding temporally structured vs. unstructured information during the second test phase, possibly reflecting a minimization of proactive interference effects. Moreover, Experiment 2 revealed significantly greater recollective recognition memory during the second test phase for temporally structured compared to unstructured encoding, suggesting that this benefit in performance may be driven by a greater ability to retrieve specific details associated with the items studied during temporally structured encoding, for example contextual (e.g., temporal and/or spatial) information. Lastly, Experiment 3 demonstrated that incidental learning can also benefit from the presentation of stimuli within a temporally structured as opposed to unstructured framework.

One possibility is that the observed benefit of temporally structured encoding is due to longer pre- or post-presentation processing time. More specifically, repeating the sequence of ISIs for initial and subsequent mini-sequence presentations in each trial would allow for some stimuli to be consistently preceded or followed by longer temporal intervals, selectively enhancing preparation or retention time. Indeed, previous work has demonstrated that recognition memory can be influenced by post-stimulus intervals, with recognition accuracy increasing as a function of post-stimulus interval duration length (e.g., Weaver, 1974; Tversky and Sherman, 1975; Intraub, 1979). Our statistical analyses did not, however, find evidence to support this (Experiment 1 Results), with accuracy not differing significantly as a function of interval lengths. Thus, in our paradigm, it is the recurring structure of the ISIs rather than their absolute length, which is critical.

It has been suggested that unpredictable stimulus onsets during encoding can have a detrimental effect on recognition memory by impacting the coordination of rehearsal and perceptual processing (Proctor, 1983). In particular, high temporal uncertainty may create interference between the rehearsal of an item and the processing of a subsequent item, leading participants to refrain from the voluntary rehearsal of stimuli (Proctor, 1983). In the current study, therefore, participants may have been less inclined to use rehearsal in the temporally unstructured encoding, leading to lower memory performance in this condition. We believe, however, that this is unlikely to account for our observed findings. Firstly, we presented stimuli in mini-sequences of 4, each repeated in succession, thereby encouraging active rehearsal during study (in both conditions) and reducing the perceptual demand of processing subsequent items. This contrasts with previous studies that have examined the impact of ISIs on recognition memory (e.g., Shaffer and Shiffrin, 1972; Proctor, 1983), in which stimuli were only presented once. Secondly, a rehearsal-perceptual processing interference account would predict a positive relationship between ISI length and recognition memory success in our temporally structured condition, a pattern that we did not observe. Finally, we observed a positive effect of a structured temporal framework during encoding on subsequent memory even when participants learned stimuli incidentally (Experiment 3).

There are a number of alternative mechanisms by which a temporally structured framework during encoding can benefit subsequent recognition memory. One possibility is that a rhythm of interval durations can be used to predict/anticipate the onset of an important event (Olson and Chun, 2001). Regular durations have been shown to promote the generating and updating of temporal expectations, which has been associated with improved sensory processing (Rohenkohl and Nobre, 2011; Rohenkohl et al., 2012) and may have a similar benefit on mnemonic processing by, for example, enhancing attention to incoming stimuli. This may be beneficial to recognition memory, as this process could lead to richer, more detailed stimulus representations and improve the discriminability of memoranda. Related to this idea is that temporal regularity, in particular how frequently stimuli occur nearby in time, has been shown to influence stimulus representations in the medial temporal lobe, including the hippocampus, even in the absence of awareness (Schapiro et al., 2012), and has been linked to subsequent memory (Smith et al., 2013).

Alternatively, consistent durations may help establish a temporal schema to which events can be anchored and learned, leading to improved subsequent retrieval. Previous work has shown that spatial and semantic schemas can facilitate the learning of new schema-consistent information (Tse et al., 2007; Van Kesteren et al., 2014). Similarly, shared contextual features can play a role in the acquisition of sequential information by promoting the associative binding of items within a sequence (DuBrow and Davachi, 2013; Ezzyat and Davachi, 2014). One possibility, therefore, is that temporal schemas operate in a similar manner, promoting the learning of new information by using contextual information related to the interval duration structure between events. Although our data do not speak to this issue, repeating interval durations may facilitate the binding of item representations within mini-sequences into a single episode during encoding, which may lead to enhanced memory. This is not inconsistent with previous research demonstrating that regularities in temporal duration can enhance the acquisition of an ordinal motor sequence (O’Reilly et al., 2008).

The results from Experiment 2 suggest that temporal regularities during learning could help to mitigate negative effects of accumulating memory interference. A key finding in recognition studies is that previously learned information can interfere with memory for new information, known as proactive interference (Criss and Shiffrin, 2004; Criss et al., 2011). A build-up of this interference can occur in which increasing the number of items tested decreases the accuracy of yes/no responses and induces forgetting (Criss et al., 2011). It is plausible that richer memory representations and/or information that is bound to a schema are less susceptible to proactive interference effects. For example, as suggested previously, temporal expectations may improve the quality of representations (Rohenkohl et al., 2012), thereby potentially increasing the discriminability of target items. Alternatively, contextual information related to the interval duration structure at encoding could facilitate the filtering of irrelevant mnemonic information (thus, limiting proactive interference from previously learned items; Jacoby et al., 2013). Although further work is necessary to investigate these suggestions, they are consistent with participants demonstrating greater recollective memory for the temporally structured task, during the second test phase.

The main finding from Experiment 3, that the benefit of temporal expectation on recognition memory can also apply to incidentally encoded stimuli is important given that everyday experiences do not always involve explicit encoding of information, and provides further support for the importance of temporally structured experiences on mnemonic processes. The findings from Experiment 3 also converge with recent fMRI work suggesting that the hippocampus may be sensitive to the interval duration information contained within sequences (Barnett et al., 2014). Using a paradigm similar to that of Experiment 3, in which participants were required to monitor interval durations between scene images across two successively presented sequences, Barnett et al. (2014) demonstrated that there was greater hippocampal activity when sequences were repeated with identical, as opposed to non-matching interval durations. Although speculative, this observation may offer potential insight into the neural mechanisms underlying the enhanced subsequent memory effect observed in Experiment 3. More specifically, the hippocampus, which has been shown to signal the passage of time between distinct events on the order of seconds (Pastalkova et al., 2008; MacDonald et al., 2011), may take advantage of the interval duration structure of events during mnemonic processing, with temporally structured events enhancing the encoding of information.

Finally, our findings raise a number of interesting questions that merit future research. For example, in the current set of experiments recognition memory was assessed immediately after study. One question, therefore, is whether increasing the length of the study-test delay (i.e., retention interval) may have an impact on the difference in recognition memory performance observed between the temporally structured and unstructured tasks. Since the present data suggest that items embedded within a structured temporal framework may be more resistant to the detrimental effects of interference, we anticipate that the observed recognition memory benefits would still be present (or perhaps be even more apparent) when the interval between study and test is increased. Further work is also necessary to provide insight into how temporal structure influences the manner in which information is encoded. For instance, as suggested above, one possibility is that temporally structured frameworks promote items within a sequence to be bound together as a single episode rather than as individual events.

## Conclusion

Our results suggest that when our experiences are underpinned by a consistent temporal structure such as a predictive rhythm, our memories for these experiences can be enhanced. Events that follow a predictable temporal structure may signal important information with regards to the environment and thus, enhancing our capacity for remembering this information may serve as an important adaptive mechanism.

## Author Contributions

ST, EO, and AL designed the experiments. ST and ZZ collected the data. ST and EO analyzed the data under the supervision of AL, and ST, EO, and AL interpreted the findings. ST, EO, and AL wrote the manuscript.

## Conflict of Interest Statement

The authors declare that the research was conducted in the absence of any commercial or financial relationships that could be construed as a potential conflict of interest.
